# Predictors of cancer patients' utilization of psychooncological support: Examining patient´s attitude and physician´s recommendation

**DOI:** 10.1007/s00432-023-05507-2

**Published:** 2023-11-17

**Authors:** Ute Goerling, Christian Albus, Corinna Bergelt, Yesim Erim, Hermann Faller, Franziska Geiser, Klaus Hönig, Beate Hornemann, Imad Maatouk, Barbara Stein, Martin Teufel, Martin Wickert, Joachim Weis

**Affiliations:** 1 Charité Universitätsmedizin Berlin, Corporate Member of Freie Universität Berlin, Humboldt‐Universität zu Berlin and Berlin Institute of Health, Charité Comprehensive Cancer Center, Charitéplatz 1, 10117 Berlin, Germany; 2 Department of Psychosomatic and Psychotherapy, Medical Faculty and University Clinic, University of Cologne, Cologne, Germany; 3 Institute of Medical Psychology, Greifswald University Medicine, Greifswald, Germany; 4 Department of Psychosomatic Medicine and Psychotherapy, University Hospital of Erlangen, Erlangen, Germany; 5 Department of Medical Psychology and Psychotherapy, Comprehensive Cancer Center Mainfranken, University of Würzburg, Würzburg, Germany; 6 Department of Psychosomatic Medicine and Psychotherapy, University Hospital Bonn, University Bonn, Bonn, Germany; 7 Department of Psychosomatic Medicine and Psychotherapy, Ulm University Clinic, Comprehensive Cancer Center Ulm (CCCU), Ulm, Germany; 8 Comprehensive Cancer Center, University Clinic Centre Dresden, Dresden, Germany; 9 Division of Medical Psychosomatics, University Hospital Würzburg, Würzburg, Germany; 10 Department of Psychosomatic Medicine and Psychotherapy, Paracelsus Medical University, General Hospital Nuremberg, Nuremberg, Germany; 11 Department of Psychosomatic Medicine and Psychotherapy, Comprehensive Cancer Center Essen (WTZ) and LVR Hospital, University of Duisburg‐Essen, Essen, Germany; 12 Comprehensive Cancer Centre, University Clinic Centre Tübingen, Tübingen, Germany; 13 Comprehensive Cancer Center, Department of Self‐Help Research, Faculty of Medicine and Medical Center, University of Freiburg, Freiburg, Germany

**Keywords:** Cancer, Utilization, Psychooncological support, Attitude, Physician´s recommendation

## Abstract

**Purpose:**

Patients with cancer suffer from a wide range of psychological distress. Nevertheless, in the literature low utilization rates of psychooncological services are reported. Various factors may influence the utilization of professional support during inpatient care. Up to now it is unclear to what extent patients’ attitude towards psychooncological support and physicians’ recommendation for psychooncological care may influence the utilization.

**Methods:**

In a multicenter longitudinal observational study in Comprehensive Cancer Centers Germany, 1398 patients with mixed cancer diagnoses were assessed at baseline during their hospital stay with respect to psychooncological distress and the need for and use of psychooncological services.

**Results:**

Psychooncological support was used by almost 28.4% of patients up to this time. A positive attitude towards psychooncological support was reported by 41.6%. A recommendation of psychooncological support by a physician was received by 16.2%. These patients reported a significant higher level of distress compared to patients who did not received a recommendation. Multivariable logistic regression detected that the utilization rate was 3.79 times higher among patients with positive attitude towards psychooncological support (OR, 3.79; 95% CI 2.51–5.73, p < 0.001). Utilization was 4.21 times more likely among patients who received a physician´s recommendation (OR, 4.21; 95% CI 2.98–5.95, p < 0.001).

**Conclusion:**

The results of the study provide evidence of the relevance of giving more attention to psychooncological distress and attitudes towards psychooncological care. To reduce reservations, patients need low-threshold information about the psychooncological services offered.

## Introduction

Although high levels of distress and up to 35% rates of mental comorbidities are reported in the literature only a moderate percentage of cancer patients demand or use psychooncological support. In Germany, a study of more than 4000 cancer patients from acute care hospitals, outpatient setting and rehabilitation clinics concluded that one in two of them is significantly distressed, and the 4 week prevalence of any mental disorder is 32% (Mehnert et al. 2018). A meta-analysis, included 1,448 cancer patients, revealed a prevalence of mental disorders in acute care ranging from 23 to 53% (Singer et al. 2010). Similar results are reported from other studies (Mitchell et al. 2011). There is a low correlation between the need measured with validated screening instruments for psychosocial distress, and the patients’ desire for psychooncological support (de Zwaan et al. 2012). In another study, the percentage of patients with an over threshold level of psychosocial distress and the subjective desire for psychoonoclogical varies between 13.7% during acute care hospital and 18.2% three months after discharge (Herbert et al. 2020). Other analyses showed a desire for support in 20% of cancer patients (Blasco, Jovell, Mirapeix, & Leon, 2022), or in 32% (Faller et al. 2016), respectively. A study of patients receiving chemotherapy showed that the desire for psychooncological support was associated with distress, anxiety, and depression (Baker-Glenn et al. 2011). Who then ultimately makes use of psychological support services? The results from the literature are different. For example, in a study of outpatient cancer patients in Canada, women, patients diagnosed with non-advanced cancer, and well-educated persons were more likely to use psychooncological support (Nekolaichuk et al. 2011). In contrast, a study of patients diagnosed with a head and neck tumor showed that patients with advanced cancer, major depression disorder, anxiety disorder, or substance use disorder were more likely to seek psychological support (Cohen et al. 2018). Nevertheless, Clover et al. (2015) reported that younger patients and women more likely reject psychooncological care. Reasons for this were “I prefer to manage myself”, “I feel supported by family or friends”, or “I feel not severely distressed”. Mosher et al. (2014) reported as barriers to seeking psychological support that 75% of patients believed they did not need it and 58% preferred to manage emotional problems by themselves. A cross-sectional study that included inpatients with different cancer diagnoses examined reasons for non-utilization of psychosocial support. These were identified as male sex, low psychological distress, perceived overload, no previous psychological treatment, and having been informed about psychosocial support (Pichler et al. 2019). The association between a good information level and the rejection of psychooncological support stands in contrast with the results of most other studies. The most apparent explanation of the authors was that the information about available psychological support during hospital stay, which was routinely offered to patients did not arouse their interest. However, most studies described an association between the lack of information about psychooncological support and the utilization (Dilworth et al. 2014; Eakin & Strycker 2001). On the other hand, a multicenter study in Germany identified several factors that led to higher utilization of psychooncological care in cancer patients. Again, these included younger age, female sex, less social support, symptoms of anxiety and depression, presence of a mental disorder, and positive attitude towards psychosocial support (Faller et al. 2017). The utilization of psychological support services by cancer patients varies greatly for a variety of reasons, including the availability of services, the patient’s age, gender, and the patient’s emotional state (Dubruille et al. 2015; Tuinman et al. 2015). Physician´s recommendation also appears to be a factor in utilization, and patient’s decision to accept or reject a support service may be influenced by a physician´s recommendation. For example, Ernst et al. (2018) report that half of patients who got a referral also received psychological support. A prospective observational study in Switzerland that examined physicians' ways of communication with cancer patients reported that a physician’s recommendation for psychooncological support significantly increased the utilization of it (Nascimento et al. 2019).

Overall, the current state of research does not provide an answer to the question of the influence of the patient's attitude toward psychooncological support.

Therefore, we address the following research aims:

- 1. What is the attitude of cancer patients towards psychooncological support?
- 2. How many patients receive a physician´s recommendation for psychooncological support?
- 3. To what extent the utilization of psychooncological support is influenced by patient´s attitude towards psychooncological support or the physician's recommendation?

## Methods

These secondary analyses are based on data from a prospective multicenter study conducted in 13 Comprehensive Cancer Centers (CCC) in Germany aimed to identify cancer patients’ needs for psychological care. All variables were systematically assessed at three measuring points: during hospitalization (baseline, t1), after 6 (t2) and after 12 months (t3). Patients were informed verbally and in writing about the study and participated after giving written informed consent. The details of the study design have been described elsewhere (Weis et al. 2018). The Ethics Committee of the University Freiburg obtained a positive approval (No. 139/13). All local ethics committees confirmed it. The study complied with the Declaration of Helsinki as well as the terms of data protection and privacy law. These analyses are focused only on data at t1 (baseline during inpatient care). All patients who had a cancer diagnosis of any site and were 18 years or older were eligible for the study. Those who did not speak German well enough, with cognitive impairments or with severe acute psychiatric or cognitive disorders were excluded.

## Measures

We used self-developed and standardized questionnaires. *Utilization of psychooncological support* was assessed by a questionnaire developed in a previous study based on patient’s self rating (Mehnert et al. 2012). The item “Have you used psychooncological support due to your cancer?” included the type of support (psychotherapy, psychological counseling).

To assess the *attitude towards psychooncological support services,* patients were asked a single question. "What is your attitude towards psychooncological support?" using a Likert scale ranging from 0 = negative to 10 = positive). In addition, we asked if their physician had recommended psychooncological support (PR) since the onset of their cancer (Response options: yes or no).

As standardized instruments we used the *Distress Thermometer.* It is a screening instrument developed by the National Comprehensive Cancer Network in the USA (2010) and validated for Germany (Mehnert et al. 2006). On a scale of 0 (= no distress at all) to 10 (= extremely distressed), the patient can indicate how distressed he or she feels. The cutoff value ≥ 5 indicates a potential need for psycho-oncological support for the patient.

*Anxiety* was measured using the General anxiety disorder-scale (GAD-7, Löwe et al. 2008). This questionnaire, based on the DSM-IV diagnostic criteria, is developed to identify patients with generalized anxiety disorders or to assess symptom severity on a four-point Likert sale rated from 0 (= not at all) to 3 (= nearly every day).

*Depression* was measured using the Patient Health Questionnaire (PHQ-9, Löwe et al. 2004). The 9-Item scale based as well on the DSM-IV diagnostic criteria for depression, and assess the severity of depressive symptoms on a four-point Likert sale rated from 0 (= not at all) to 3 (= nearly every day).

Sociodemographic data were assessed by patients’ self-reports. Medical information were taken from electronic medical records.

## Statistical analyses

First, descriptive statistics were calculated for the sample description and the items about patients' attitude towards psychooncological support and whether a recommendation for such support was received. Furthermore, associations between categorical variables were analyzed by using chi^2^-test, and associations between continuous variables by using Pearson correlation. Missing values were excluded and no imputation techniques were used. For further analyses regarding attitude, the responses were divided into three categories (0–3 = negative, 4–6 = neutral, 7–10 = positive). For statistical analyses we used for GAD-7 the cut-off score of ≥ 10 as threshold for lower or higher level of anxiety (Andersen et al. 2014). The same procedure was used for the PHQ-9. To analyze a possible influence of attitude and PR on utilization of psychooncological support, we used multivariable logistic regression adjusted for age, sex, distress, anxiety, and depression. In this procedure, missing data were deleted listwise. Odds ratio (OR) for utilization of psychological support was estimated with 95% confidence interval (95% CI), and tested using the Wald Test. A p-value < 0.05 was considered as statistically significant. Analyses were performed with SPSS version 28.0.1.0 (SPSS Inc., Chicago, Ill., USA).

## Results

### Sample characteristics

Of a total of N = 6088 oncology patients treated at the respective organ cancer centers during the recruitment period of 9 months, N = 3046 (50%) were asked by study personnel to participate in the survey at time t1. of these, N = 1632 (53.6%) participated. Recruiting rate differed across the participating centers. On average, 108 patients participated per CCC (for details see Weis et al. 2018). After reducing missing data regarding the target variables, the data set included 1,398 participants. Mean age of the sample was 59 years (SD = 12.1) and 58.3% were women. The sample includes patients with different tumor entities (Table 1). A detailed description can be found in Weis et al. (2018).

**Table 1:** Sociodemographic and medical characteristics of the sample (N = 1398)

Mean age (years)	59 (SD = 12.1)	
Sex	N	%
Female	812	58
Male	577	42
Cancer type		
Gastrointestinal	228	16.3
Breast	286	20.4
Melanoma	214	15.3
Gynecological	197	14.1
Lung	122	8.7
Male genital, prostate	83	5.9
Head and neck	81	5.8
Hematological	71	5.1
Sarcoma	18	1.3
CNS neoplasia	8	0.6
Urological	8	0.6
Others	53	3.8
Metastases		
No	732	52.4
Yes	666	47.6

*SD* standard deviation

A clinically relevant distress score with a cutoff ≥ 5 was detected for 59% of the patients. Furthermore, 13.6% show elevated level of anxiety (GAD-7 ≥ 10), and 22.3% elevated level of depression (PHQ-9 ≥ 10).

### Patients attitude towards psychooncological support

In terms of their attitude towards psychological support, a positive attitude reported by 551 patients (41.6%), while 285 (21.5%) reported a negative one. A neutral attitude was reported by 487 patients (36.8%). Women reported a positive attitude more frequent compared to men (66.4% vs. 33.6%). With respect to age, patients aged 46 to 55 years (30.7%) and 56 to 65 years (32.2%) were more likely to have a positive attitude compared to patients under 45 years (16.1%).

### Physician´s recommendation (PR) for psychooncological support

A recommendation for psychooncological support by their physician (PR) received n = 226 patients (16.2%). Compared with the group without PR the mean distress score is significantly higher (M = 5.45, SD = 2.28 vs M = 4.77, SD = 2.49; p < 0.001). The proportion of patients with clinically relevant distress (cutoff ≥ 5) is also higher in the group that received a PR (68.6% vs 31.4%, p < 0.01) compared to the group without distress and received PR. Regarding anxiety and depression, 25.2 and 34.5% of patients, respectively, reported a score above a cut-off of 10 to have received a recommendation from their physician (Table 2).

**Table 2:** Proportion of patients over threshold with respect to physician’s recommendation for psychooncological support

	Distress^a^ Cut-off ≥ 5	Anxiety^b^ Cut-off ≥ 10	Depression^c^ Cut-off ≥ 10
Physician’s recommendation for psychooncological support n = 226	68.6%	25.2%	34.5%

^a^measured with the Distress Thermometer

^b^measured with General Anxiety Disorder 7 (GAD-7)

^c^measured with Patient Health Questionnaire (PHQ-9)

Out of all cancer patients 397 patients (28.4%) state that they had used a psychooncological support during inpatient care at the time of survey (t1). Utilization shows correlations with distress, anxiety, depression, attitude, and physician´s recommendation (PR) (Table 3).

**Table 3:** Correlation between utilization of psychooncological support, distress, anxiety, depression, attitude towards psychooncological support and physician´s recommendation for psychooncological support (Pearsons´ correlation)

	Mean (SD)	Utilization of psychological support	Distress^a^	Anxiety^b^	Depression^c^	Attitude^d^
Utilization of psychological support						
Distress^1^	4.90 (2.484)	0.117**				
Anxiety^2^	4.67 (4.332)	0.229**	0.510**			
Depression^3^	6.38 (4.761)	0.189**	0.496**	0.732**		
Attitude^4^	5.75 (2.890)	0.261**	0.005	0.083**	0.033	
Physician´s recommendation for psychological support		0.299**	0.102**	0.188**	0.164**	0.165**

^a^measured with the Distress Thermometer

^b^measured with General Anxiety Disorder 7 (GAD-7)

^c^measured with Patient Health Questionnaire (PHQ-9)

^d^patients’ attitude towards psychooncological support

*SD*  Standard deviation

**The correlation is significant at the 0.01 level (2-sided)

### Multivariate logistic regression

To analyze a possible influence of attitude and PR on utilization of psychooncological support, we created three models by using multivariable logistic regression. For each model in the first step unadjusted, in the second step adjusted for age, sex, distress, anxiety, and depression. In this process, missing data was deleted listwise. In the first model (M1), the variable of attitude was first tested unadjusted and then adjusted for the named variables. In the second model (M2), the above mentioned two steps were performed with the variable PR. The third model (M3) combines the two variables. It tests attitude and PR first together unadjusted and in the second step including the adjustment variables.

Multivariate logistic regression analyses adjusted for age, sex, distress, anxiety, and depression show significantly higher OR for utilization of psychooncological support for patients with positive attitude (OR, 3.79; 95% CI, 2.51–5.76; p < 0.001; Model 1). In the same multivariate logistic regression analyses adjusted for age, sex, distress, anxiety, and depression significant higher OR were found for utilization of psychooncological support among patients who got PR for it (OR, 4.21; 95% CI, 2.98–5.95; p < 0.001; Model 2). In Model 3 utilization of psychooncological support, adjusted for the named variables, was 3.54 times higher for patients with positive attitude compared to negative attitude (OR, 3.54; 95% CI, 2.26–5.27, p < 0.001). If patients had received a PR utilization was 3.72 times higher (OR, 3.72; 95% CI 2.59–5.33, p < 0.001) compared to patients, who not received PR. All results listed in Table 4.

**Table 4:** Odds ratios for utilization of psychooncological support

Utilization of psychooncological support									
			Unadjusted				Adjusted^a^		
		OR	Lower 95% CI	Upper 95% CI	p Value	OR	Lower 95% CI	Upper 95% CI	p Value
*M1*									
Attitude	Negative	Ref				Ref			
	Neutral	1.52	1.04	2.23	0.032	1.32	0.85	2.02	0.216
	Positive	4.18	2.91	5.99	< 0.001	3.79	2.51	5.73	< 0.001
			Nagelkerke R^2^ 0.097, n = 1323				Nagelkerke R^2^ 0.289, n = 1285		
*M2*									
PR		4.93	3.66	6.65	< 0.001	4.21	2.98	5.95	< 0.001
			Nagelkerke R^2^ 0.112, n = 1374				Nagelkerke R^2^ 0.290, n = 1334		
*M3*									
Attitude	Negative	Ref				Ref			
	Neutral	1.53	1.03	2.26	0.035	1.35	0.87	2.09	0.185
	Positive	3.57	2.46	5.18	< 0.001	3.54	2.26	5.27	< 0.001
PR		4.12	3.02	5.62	< 0.001	3.72	2.59	5.33	< 0.001
			Nagelkerke R^2^ 0.173, n = 1306				Nagelkerke R^2^ 0.334, n = 1269		

p Value, significance level

*M1* model 1, *M2* model 2, *M3* model 3, *PR* physician´s recommendation for psychological support, *OR* odds ratio, *95% CI* 95% confidence interval of odds ratio, *Ref* reference group

^a^Adjusted for age, sex, distress, anxiety, and depression

## Discussion

The purpose of this secondary analysis was to examine the influence of patients´ attitude towards psychooncological support and physician´s recommendation (PR) for psychooncological support on cancer patients' utilization of psychooncological support. More than half of the patients reported a clinically relevant distress level, which is in line with results from most studies in the literature (Meggiolaro et al. 2016; Mehnert et al. 2018). National and international cancer guidelines recommend appropriate support to help patients manage their disease and improve their quality of life. Although the effectiveness of psycho-oncological interventions has been proven (Faller et al. 2013; Kalter et al. 2018), not all patients with high levels of psychosocial distress seek for psychooncological support. Various reasons for this in particular organizational issues, feeling enough support by partner or family or others have been described in the literature (Dilworth et al 2014). For example, Faller et al. (2017) reported that 13.4% of patients reported having sought psychooncological support because of distress due to their cancer. In another study with a large sample of cancer patients from 13 German comprehensive cancer centers with various tumor sites in the inpatient setting, 28.4% reported having used professional psychooncological support independent of measured distress (Weis et al. 2018). This result is in line with other studies (Cohen et al. 2018), that reported utilization in patients around 29%. Approval as a certified Comprehensive Cancer Center in Germany requires the availability of psychooncological services. Annual reports show a wide range of utilization in the various centers between 15% to more than 50% (https://www.onkozert.de/2023/09/15/jahresberichte-der-zertifizierungssysteme-2023/).

In our sample about 40% reported a positive attitude towards psychooncological support. Most of them were women. It is known that women are more likely to report psychological problems and distress than men, and it could also be that women are more open to psychooncological support services. Our results suggest that the likelihood of seeking psychological support is 3.79 times higher in patients with a positive attitude. A higher likelihood of using support services in connection with a positive attitude has also been shown in other studies (Faller et al. 2017; Steginga et al. 2008). A German study investigated the relationship between patients’ positive or negative attitudes towards seeking help after cancer and psychooncological support utilization. Positive attitude explained most variance of cancer patients’ current psychooncological support utilization (Leppin et al. 2019). A systematic review about patient and health professional’s perceived barriers to utilization of psychooncological support identified no need for psychooncological support, lack of information about services including knowing that services exist, and also negative attitude among others (Dilworth et al. 2014). A negative attitude could be the result of experience, but also of concern about further stigmatization, or a fundamentally negative attitude towards outside help.

The proportion of patients who received physician´s recommendation (PR) for psychooncological support is very low at 16.2%. It may well be discussed at this point that patients are not always sure for which supportive specialty recommendations have been made by physicians. After all, there seems to be at least a positive correlation with the distress here, though not for anxiety and depression. Several points need to be discussed here. On one hand, it has been documented that physicians do not adequately recognize patients' distress and that patients do not always communicate their distress (Fritzsche et al. 2004). On the other hand, there was no explicit care pathway for psychooncological support defined in the 13 centers of this study. Therefore, it could be the case that the results of the questionnaires on distress, anxiety, and depression collected for this study, were not yet available for the physician at the time of the medical consultation. However, our analyses suggest that PR increases the likelihood of utilization of psychooncological support by a factor of 4.21. In an Australian study, the utilization rate of psychooncological services by survivors of gynecologic cancer was increased a nearly 12-fold by PR (Beesley et al. 2010). This enormous difference can be explained primarily by the different study designs, and the specific tumor entity.

Even the variant calculated in Model 3, that the patient has a positive attitude and a PR, showed an increase in the probability of utilization of more than threefold. Since attitude towards psychological support also depends on experience, results support that patients who have already undergone psychotherapy are more likely to seek psychooncological support (Pichler et al. 2019; Weis et al. 2018). It would be interesting to investigate the attitude towards psychooncological support not only in patients but also in physicians, nurses and other healthcare providers. I.e., to ask if they were convinced of the positive effects of supporting interventions and if they were familiar with the content of the work done by psychological colleagues. Then they would be able to recommend them more often and with more vigor. Communication training could help reduce uncertainties.

### Study limitations

The strength of the study lies in its large sample size with various tumor diagnoses, recruited in all CCCs across Germany. Nevertheless, there are some limitations. As in other studies of patients with severe physical illness, there was a high rate of refusal to participate in the study (46.4%), may be due to severe physical burden or psychological distress. Unfortunately, we were not able to assess why the patients did not give consent to participate in our study. It could be assumed that more patients with a positive attitude participated in this study. Moreover, the results may not be generalizable to tumor patients in general because we recruited only in CCCs and the sample sizes and tumor entities varied among CCCs,.

### Clinical implications

The results of our analyses were focused on the influence of the patients’ attitude towards psychooncologcal support and its utilization. Regarding the patients' attitude towards psychooncology, low-threshold information about psychooncological support and the goals of supportive care are necessary. This information should be also provided by the physicians. Asking the patients for negative experiences with psychosocial support in their cancer history may be helpful for understanding reasons for rejection of psychooncological support. The screening for distress and the communication with the patient should be an integral part of an interdisciplinary oncology. More information is needed about additional factors influencing the utilization of psychooncological support.

## Data Availability

The datasets generated during and/or analysed during the current study are available from the corresponding author on reasonable request.
